# Bone Mineral Density in Patients with Hepatic Glycogen Storage Diseases

**DOI:** 10.3390/nu13092987

**Published:** 2021-08-27

**Authors:** Jésica Tamara Jacoby, Bruna Bento dos Santos, Tatiele Nalin, Karina Colonetti, Lília Farret Refosco, Carolina F. M. de Souza, Poli Mara Spritzer, Soraia Poloni, Roberta Hack-Mendes, Ida Vanessa Doederlein Schwartz

**Affiliations:** 1 Graduate Program in Medical Sciences Medicine, Department of Medical College, Universidade Federal do Rio Grande do Sul, Porto Alegre 90040-060, Brazil; nutricionista.jesica@gmail.com; 2 Graduate Program in Genetics and Molecular Biology, Department of Genetics, Universidade Federal do Rio Grande do Sul, Porto Alegre 90040-060, Brazil; bruna.bdsantos@hotmail.com (B.B.d.S.); karinacolonetti@hotmail.com (K.C.); 3 Basic Research and Advanced Investigations in Neurosciences (BRAIN) Laboratory, Hospital de Clínicas de Porto Alegre, Porto Alegre 90035-903, Brazil; tatinalin@gmail.com (T.N.); soraiapoloni@yahoo.com.br (S.P.); 4 Ultragenyx Brasil Farmacêutica Ltda, São Paulo 07093-080, Brazil; 5 Medical Genetics Service, Hospital de Clínicas de Porto Alegre, Porto Alegre 90035-903, Brazil; lrefosco@hcpa.edu.br (L.F.R.); cfsouza@hcpa.edu.br (C.F.M.d.S.); 6 Department of Genetics, Universidade Federal do Rio Grande do Sul, Porto Alegre 90040-060, Brazil; 7 Division of Endocrinology, Hospital de Clinicas de Porto Alegre, Porto Alegre 90035-003, Brazil; spritzer@ufrgs.br; 8 Department of Physiology, Universidade Federal do Rio Grande do Sul, Porto Alegre 90050-170, Brazil; 9 School of Agriculture and Food Science, University College Dublin, D04 V1W8 Dublin, Ireland; roberta.hackmendes@ucd.ie; 10 Nuclimed, Center for Clinical Research, Hospital de Clínicas de Porto Alegre, Porto Alegre 90035-903, Brazil

**Keywords:** glycogen storage disease, bone mineral density, GSD, bone, osteocalcin, photon absorptiometry

## Abstract

The association between bone mineral density (BMD) and hepatic glycogen storage diseases (GSDs) is still unclear. To evaluate the BMD of patients with GSD I, IIIa and IXα, a cross-sectional study was performed, including 23 patients (GSD Ia = 13, Ib = 5, IIIa = 2 and IXα = 3; median age = 11.9 years; IQ = 10.9–20.1) who underwent a dual-energy X-ray absorptiometry (DXA). Osteocalcin (OC, *n* = 18), procollagen type 1 N-terminal propeptide (P1NP, *n* = 19), collagen type 1 C-terminal telopeptide (CTX, *n* = 18) and 25-OH Vitamin D (*n* = 23) were also measured. The participants completed a 3-day food diary (*n* = 20). Low BMD was defined as a Z-score ≤ −2.0. All participants were receiving uncooked cornstarch (median dosage = 6.3 g/kg/day) at inclusion, and 11 (47.8%) presented good metabolic control. Three (13%) patients (GSD Ia = 1, with poor metabolic control; IIIa = 2, both with high CPK levels) had a BMD ≤ −2.0. CTX, OC and P1NP correlated negatively with body weight and age. 25-OH Vitamin D concentration was decreased in seven (30.4%) patients. Our data suggest that patients with hepatic GSDs may have low BMD, especially in the presence of muscular involvement and poor metabolic control. Systematic nutritional monitoring of these patients is essential.

## 1. Introduction

Glycogen storage diseases (GSDs) are characterized by abnormal storage or catabolism of glycogen due to the deficient activity of enzymes that catalyze glycogen synthesis or degradation [1]. Their incidence is approximately 1 in 20,000–43,000 live births worldwide. About 80% of hepatic GSDs are of types I, III and IX [2]. GSD I is caused by deficient activity of glucose-6-phosphatase [3,4]. GSD III results from a deficiency of glycogen debranching enzyme [5,6], and it is classified in IIIa, which causes liver and muscular compromise, and IIIb, which causes only liver symptoms [7]. GSDIXα, caused by pathogenic variations in the phosphorylase kinase alpha 2 (*PHKA2*) gene, results from the absence of the liver-α subunit of the phosphorylase kinase, which causes liver symptoms only [8,9].

The treatment of hepatic GSDs usually consists of dietary management, with frequent periodic administration of uncooked cornstarch (UCCS) and restriction of simple carbohydrate intake [10]. Dietary management aims to maintain normoglycemia, preventing secondary metabolic derangement and the development of complications such as hepatocellular adenomas/carcinomas, myopathy, renal failure and osteoporosis [11]. In GSD I, the restriction of sucrose, fructose, galactose and lactose is widely used; in GSDs III and IX, the restriction of sucrose and the intake of a high-protein diet are also common [4,6,12]. Other strategies for treatment are described, such as the continuous infusion of glucose at night, ketogenic diet [10,13], medium chain triglycerides (MCTs) [14,15] and liver transplant [2].

Low bone mineral density (BMD) in GSD patients and a higher risk of developing fractures have been described by several studies [16,17,18,19,20,21]. Its etiology seems to be multifactorial, a combined result of malnutrition, metabolic imbalance, secondary lactic acidosis and hypogonadism [16,18]. Other inborn errors of metabolism (IEM) have described BMD within normal limits, but lower than the population reference [22,23].

The uncoupling of bone turnover is associated with bone loss, increased fracture risk and poor adherence to the treatment of osteoporosis [24]. It is important to point out that there are many determinants of the variability of bone turnover, such as age and gender. Moreover, there are conditions characterized by an acceleration of bone turnover, such as primary hyperparathyroidism and vitamin D deficiency [25]. The bone formation marker procollagen type 1 N-terminal propeptide (P1NP) and the resorption marker collagen type 1 C-terminal telopeptide (CTX) are the preferred markers for evaluating bone turnover in the clinical setting due to their specificity to bone, performance in clinical studies, wide use and relatively low analytical variability [24]. Some studies have correlated BTMs with hepatic GSD and treatment variables, and those few studies have reported inconclusive findings [16,18,21,26].

The main aim of this study was to evaluate the BMD of patients with GSD I, IIIa and IXα by dual emission X-ray absorptiometry (DXA).

## 2. Materials and Methods

This was a cross-sectional outpatient study carried out at Hospital de Clínicas de Porto Alegre (HCPA), Brazil. The study was approved by the HCPA Research Ethics Committee (protocol numbers 14-0120 and 15-0218) and conducted in accordance with the Declaration of Helsinki [27]. All participants (or their legal guardians where appropriate) provided written informed consent.

### 2.1. Subjects

The study included 23 patients with previous diagnosis of GSD (Ia = 13, Ib = 5, IIIa = 2, IXα = 3), confirmed by genetic analysis and recruited from the Medical Genetics Service of HCPA (MGS-HCPA), Brazil; 12/23 were female, with a median age of 11.9 years, ranging from 3 to 33 years (0 to 11 years, *n* = 12; 12 to 19 years, *n* = 5; ≥20 years, *n* = 6). The treatment protocol for patients with GSD I seen at the MSG-HCPA includes restriction of sucrose, fructose, galactose and lactose, in addition to regular consumption of UCCS; for types III and IX, restriction of sucrose, the intake of UCCS and a high-protein diet are also recommended.

### 2.2. Data Collection

#### 2.2.1. DXA Analysis

BMD was assessed by DXA using a Lunar Prodigy Primo device (Encore version 14.1, Radiation Corporation, Madison, WI, USA). Patients fasted for only 2 h before the scan, as they cannot tolerate longer periods without presenting hypoglycemia. In adults, the lumbar spine (L1–L4) and proximal femur (total femur and femoral neck) were evaluated, while in children, the lumbar spine (L1–L4) and whole body (minus the head) were assessed. Precision error was assessed as recommended by the International Society for Clinical Densitometry (ISCD), taking into account the minimum acceptable precision for an individual technologist [28,29].

As per the 2019 ISCD position statement, results were expressed as Z-scores for age. In “BMD below the expected range for age”, we observed a Z score of −2.0 standard deviations (SDs) or lower [28,29], and this was the criterion adopted for classifying the BMD as low or reduced in this study.

Patients with short stature for age did not have their height corrected for DXA to avoid false normalization of BMD in patients whose height deficits may be due to the underlying GSD.

#### 2.2.2. Biochemical Analysis

On the day of the DXA scan, a blood sample was collected after the same 2 h fasting. Serum was separated from the sample, stored at –80 °C, and sent to a reference laboratory (Fleury S.A., São Paulo, Brazil), where commercially available kits (Roche Diagnostics, coefficient of variation 5%) were used to quantitate three BTMs: osteocalcin (OC), P1NP and CTX by electrochemiluminescence. The reference ranges for these BTMs were classified by age and sex according to Bayer et al. (2014) and Wyness et al. (2013) (Table S1) [30,31]; in the present study, the values of BTMs were considered abnormal if they were 20% above or below the upper or the lower limit of the reference range, respectively. 25-OH Vitamin D concentrations were also measured (NRV = 20ng/mL).

Additional test results, such as CPK, vitamin B12, calcium, phosphorus, triglycerides, cholesterol (total and fractions), glucose, lactate and arterial blood gas, were collected from the medical records of each patient on the date closest to (up to 20 days before or after) the DXA scan. For lactate and glucose, the median of all values available for the 24 months preceding DXA was also described. 

Serum triglyceride (the measurement closest to the date of DXA), glucose and lactate concentrations (the median value of the measurements of the previous 24 months) were used to determine the metabolic control of patients [21,26,32]. Patients were considered adherent to treatment (i.e., with a good metabolic control) if the values for all three variables were within the normal reference range (glucose: 4.2–5.6 mmol/L; lactate: 0.5–2.2 mmol/L; triglycerides: <2.3 mmol/L).

#### 2.2.3. Food Diary

Patients completed a 3-day food intake diary. This was completed before the DXA scan and included 2 weekdays and 1 weekend day. Patients were given detailed instructions on how to record their food intake, including recording food preparation and product brands. Analysis was carried out using Nutribase software (NB16Cloud, CyberSoft, Inc., Phoenix, AZ, USA). Household measurements were converted to grams using the 2008–2009 Brazilian Household Budget Survey as a basis. The results were compared with DRI and the recommended guidelines for GSDs I, III and IX, with a 10 to 15% protein target for GSD I, 20 to 30% in GSD III and 20 to 25% of total calories in GSD IX. The daily energy requirement was calculated using the Harris Benedict formula [4,6,10,12,33].

#### 2.2.4. Anthropometric Measurements

On the same day that DXA was performed, weight was measured on a digital scale (0.1 kg resolution; Model 2096PP/2; Toledo, São Paulo, Brazil), and height was measured with a wall-mounted Harpenden stadiometer (0.1 cm accuracy; Holtain Ltd., Crymych, Wales, UK). Measurements were made with the participants standing upright, wearing lightweight clothing. The body mass index (BMI) was calculated as the weight (kg) divided by height (m) squared, and classified as underweight, eutrophic, overweight or obese, according to the World Health Organization (WHO) criteria. In patients aged under 19, nutritional status was calculated using BMI for age, with Z-scores from WHO Anthro version 3.2.2 and WHO AnthroPlus version 1.0.4 [34,35].

### 2.3. Statistical Analysis

Categorical variables were expressed as absolute and relative frequencies, and continuous variables as medians, standard error, interquartile range and percentiles. The Kruskal–Wallis test was used to compare groups and the Spearman test for correlations. All statistical analyses were performed in IBM SPSS Statistics for Windows, Version 19.0 (IBM Corp., Armonk, NY, USA). For correlations, the significance level was set using Bonferroni correction at *p* ≤ 0.004.

## 3. Results

### 3.1. Characteristics of the Sample

The clinical and laboratory findings of the sample are shown in Table 1. The data were stratified by age group (children, aged 0 to 11; adolescents, aged 12 to 19; and adults, aged 20 or older). All patients were on UCCS therapy (4 to 8 times a day, median dose = 6.3 g/kg/day) and no patient received additional therapies such as MCT, ketogenic diet or liver transplant. Eleven patients (47.8%) met the criteria for good biochemical metabolic control (children = 6/12, adolescents = 1/5, adults = 4/6) (Table S2). All patients with GDS IIIa had high CK levels. No patient presented metabolic acidosis.

Nine out of the 12 children (75.0%) were overweight or obese. Adolescents (*n* = 4/5) and adults (*n* = 4/6) were also overweight, with a median BMI of 29.0 kg/m² and 27.7 kg/m², respectively.

Overall, 4/12 children (patients 5, 15, 21 and 22) and 3/6 adults (patients 9, 12 and 23) could be classified as 25-OH Vitamin D-deficient (Tables S2 and S3). Of these, only one patient was not on a multivitamin or Vitamin D supplement.

### 3.2. DXA

Three patients presented a BMD Z-score ≤ −2.0: a 6.6-year-old GSD Ia, and two GSD IIIa patients aged 11.9 and 16.5 years (Tables S3 and S4; patients 1, 19 and 20). All had adequate 25-OH Vitamin and BTM status (Tables S3 and S4). Only the GSD Ia patient had a high lactate concentration (5.8 mmol/L). Two of these patients (the GSD Ia, and the GSD IIIa 11.9 years) had a height Z-score below the reference range (patients 1 and 19, Tables S2–S4).

### 3.3. BTMs and Correlations with DXA, Metabolic Control and Biochemical Parameters 

BTMs were evaluated in 19 patients (children = 10; adolescents = 3; adults = 6). Overall, BTM levels decreased with age, as would be expected in the normal population (Table S1). CTX correlated positively with P1NP (r = −0.814, Table S5). There was a negative correlation between body weight and BTMs (CTX, r = −0.652; P1NP, r = −0.690; OC, r = −0.668; Table S5). The difference of BTMs between types of GSD was not significant (data not shown).

According to the dietary data, the energy intake median adequacy ratio was moderately to strongly associated with the bone biomarkers OC and P1NP (Table S5).

Only three female patients (patients 8, 9 and 11), ranging from 19 to 24 years, presented high levels of P1NP (Table S3). Among them, only patient 9 was 25-OH Vitamin-deficient.

### 3.4. Nutritional Adequacy

**Figure 1:**
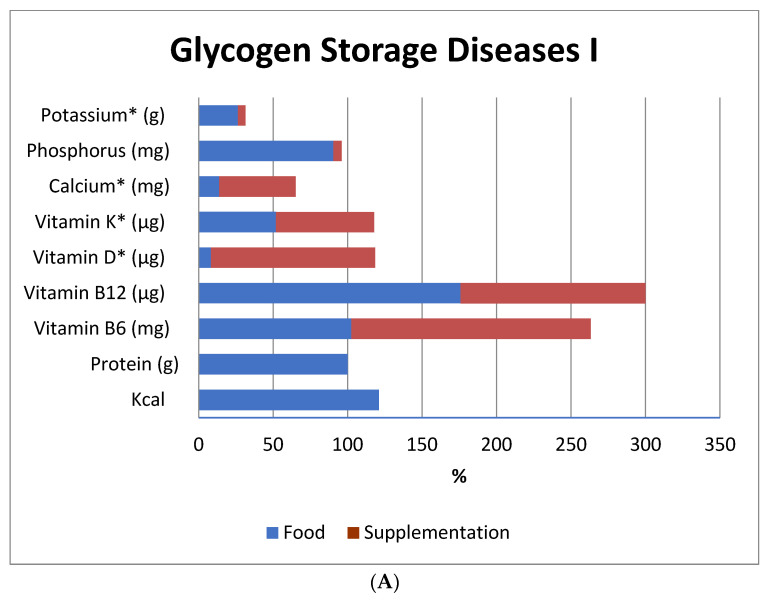

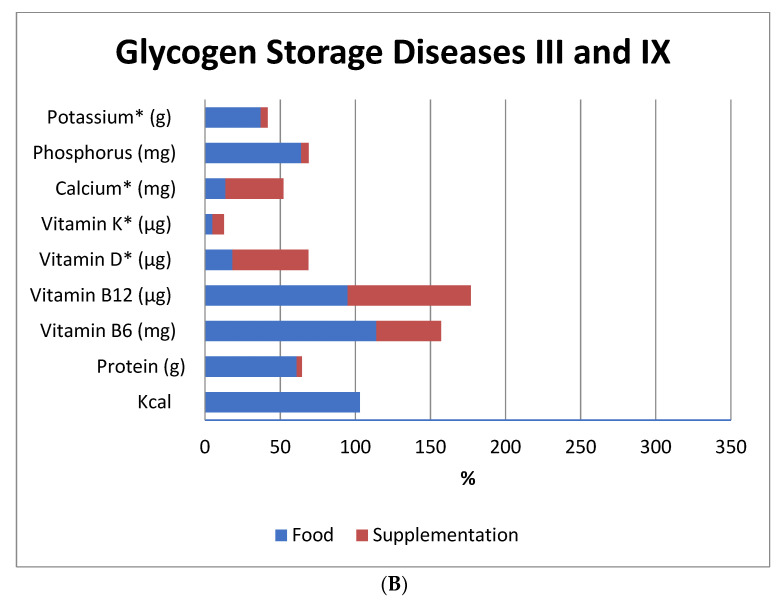
Adequacy of dietary and supplemental intake of nutrients essential for bone metabolism. (**A**) Data for Glycogenosis Ia (*n* = 11) and Ib (*n* = 5) patients. (**B**) Data for Glycogenosis IIIa (*n* = 1) and IXα (*n* = 3) patients. Three patients did not complete the food diary. Data expressed as median; adequacy expressed as % of RDA or * AI. Data obtained through food record; adequacy according to GSD treatment guidelines (for protein) and DRI tables (all other nutrients).

Figure 1 illustrates the adequacy of nutrient intake (from the diet and with the addition of prescribed dietary supplements) in the sample.

#### 3.4.1. Energy Intake

Energy intake was higher than the recommended intake in GSD Ia and Ib (121.0% adequacy of the recommended energy requirement). In GSDs IIIa and IXα, energy intake met the reference intakes (104.0%) (Table S6).

#### 3.4.2. Protein Intake

Protein intake was less than the recommended intake for GSDs IIIa and IXα (4/5; 1.6 g/kg/d) (Table S6).

#### 3.4.3. Vitamin and Mineral Intake

Adequate vitamin D intake was only achieved after supplementation, except for GSD IXα patients. Diet and supplementation failed to achieve adequate intakes of vitamin K, calcium, potassium and phosphorus. 

Nutritional supplementation (multivitamin, calcium or vitamin D) was reported by 20/23 of the patients. There was no association between supplement use, BTMs and BMD.

## 4. Discussion 

In this study, only 13.0% (*n* = 3) of the subjects presented a clinically low BMD, two subjects with GSD IIIa and one with GSD Ia. All the other children presented a BMD within the normal parameters. This finding is in contrast to previous studies [16,17,18,20,21,26,36] reporting a higher clinical incidence of low BMD in hepatic GSD. Of the three subjects identified with a low BMD, two had GSD IIIa, involving muscular weakness, and the GSD Ia had a high lactate concentration. Both are associated with poor bone mineralization. 

A summary of the findings of the present study and a comparison with previous studies in the literature on DXA and hepatic GSD are shown in Table S7. The differences observed between our cohort and other cohorts regarding the prevalence of low BMD may be due to the younger age and the higher BMI of our sample. Moreover, there are differences in DXA site (lumbar only) [21,26]; in cutoff points for osteopenia and osteoporosis (SD: −1.0 and −2.5, respectively) [16,21]; and in dietary therapy (normal diet or overnight enteral glucose feeding [16,20] and lower UCCS dose [18,21] as compared to our patients). 

In addition to these differences, we found that levels of triglycerides [18,20,21,26], total cholesterol [18,21,26] and lactate [20,21] were lower than those reported by previous studies, suggesting a better metabolic control in our sample.

The interpretation of the values of BTMs represents a challenge, especially in young patients [30]. BTMs are high in infancy, decrease in childhood and then increase during puberty. The use of an appropriate reference range for age groups is therefore mandatory [25]. In our study, BTM values decreased with age, as would be expected in the normal population.

Other authors have demonstrated increased bone remodeling and/or reduced bone deposition in patients with GSDs I and III through the analysis of calcitonin, CTX, N-terminal telopeptide of type 1 collagen (NTX), hydroxyproline and OC [18,21]. On the other hand, Cabrera-Abreu et al. (2004) measured type 1 collagen terminal propeptide (P1CP), P1NP, CTX and bone alkaline phosphatase (ALP) levels and found no correlation with BMD in patients with GSDs I, III or IX [16]. We found high levels of P1NP in only three female patients, all above 19 years and with normal BMD. For one patient, the increase in P1NP could have been due to vitamin D deficiency. Unfortunately, we have not evaluated other causes of increased BTM, such as hyperparathyroidism and thyrotoxicosis.

The negative correlation between body weight and OC, P1NP and CTX is a finding that deserves more investigation. The relationship between BMI and BMD has already been reported; higher BMI is associated with lower prevalence of osteopenia and osteoporosis [37,38]. Studies of Brazilian healthy males (adolescents and young adults) also demonstrated a positive correlation between BMI and BMD [39,40]. Conversely, reductions in body weight were associated with decreased bone buildup in adolescents [41]. This relationship between higher weight and better BMD may be attributable to a larger mechanical load on bones [38], or to the hypothesis that adipocytes may be hormone-producing cells and thus may interfere directly or indirectly with the activity of osteoclasts and osteoblasts [42].

In our study, we also observed a negative association between adequacy of energy intake and OC and P1NP. The correlation between caloric intake and OC concentrations is not well described; however, energy expenditure appears to be greater when OC is increased. Studies with animal models have found that higher concentrations of OC are associated with lower odds of developing obesity, as the increased energy expenditure leads to less buildup of fat and body mass [43,44]. 

Regarding biochemical data, we observed a median of 25-OH Vitamin D concentration, lower than those found by Kaiser et al. (2019) and Melis et al. (2014). Like the aforementioned authors, we found no association between 25-OH Vitamin D levels and BMD [20,21]. Despite the reduced consumption of vitamin D in their diet, few patients had low 25-OH Vitamin D concentrations, probably because food consumption does not necessarily reflect serum concentrations. The main source of vitamin D is endogenous synthesis after skin exposure to UVB radiation [45].

We also observed a low intake of nutrients important for bone metabolism, even considering that about half of the patients had poor metabolic control. The main sources of nutrients supporting bone health are dairy products, as they provide a good supply of calcium, phosphorus, potassium, protein and vitamin D [46]. Patients with GSD I have very strict restrictions on sucrose, lactose and fructose intake [4], which makes it difficult for them to reach their nutritional goals. In GSDs III and IX, intake of these sugars is not as restricted as in I, but remains limited [6,12]. Our data suggest that poor metabolic control, as defined herein, does not necessarily reflect a higher intake of the controlled food. 

A healthy and varied diet is the optimal means of achieving nutritional goals, since nutrients from food are safer and more bioavailable than those from supplements; however, the UCCS therapy and dietary restrictions imposed on patients with hepatic GSDs make this a difficult task. Thus, treatment guidelines for GSD I systematically recommend supplementation with calcium and multivitamins [4,10]. The use of low-dose calcium supplementation is justified by of the risk of kidney stone formation due to hypocitraturia in patients with GSD I [47]. For GSDs III and IX, recommendations are individualized on the basis of the nutritional needs and dietary intake of each patient [6]. As we observed a similarly insufficient nutrient intake profile in the two groups, our findings suggest that it would be prudent to assess the renal function of patients and then recommend supplementation in both groups. It is important to highlight that intestinal calcium absorption may be influenced by the gut microbiota [48,49,50]. Colonetti et al. (2019) and Ceccarani et al. (2020) described that patients with GSD presented intestinal dysbiosis and increased pro-inflammatory rate. This can indicate a higher risk for low BMD, which was not confirmed by our study [50,51,52].

According to the DRI, intake of energy, vitamin B6 and vitamin B12 exceeded the daily recommendations. Previous studies, with older populations, have noted that excessive intake of vitamins B6 and B12 is associated with an increased risk of hip fracture [53,54]. We suggest that the association between bone health and intake of B vitamins in patients with hepatic GSDs be investigated in future studies.

This study has some limitations, such as the small and heterogeneous sample, the lack of data on the Tanner scale and physical activity level, and the reliance on self-reported food records (which may under- or overestimate actual dietary intake).

## 5. Conclusions

Our data suggest that BMD is clinically normal in most patients with hepatic GSDs, especially when they present high BMI and good metabolic control. However, it seems to be different for GSD III patients; they are prone to having bone health issues even with high BMI and good metabolic control. It will be important to develop additional studies to analyze the beneficial effects of physical activities on this cohort. 

## Figures and Tables

**Table 1:** Clinical and laboratorial characteristics of patients with hepatic glycogen storage diseases included in the study (*n* = 23).

Variables	Overall (*n* = 23)	Children (*n* = 12)	Adolescents (*n* = 5)	Adults (*n* = 6)	Reference Range
**Sex (F/M)**	12/11	4/8	3/2	5/1	-
**GSD type (Ia/Ib/IIIa/IXα)**	(13/5/2/3)	(6/3/1/2)	(3/1/1/0)	(4/1/0/1)	-
**Weight (kg)**	56.0 (38.8–72.0)	38.9 (28.5–46.5)	76.0 (66.0–79.0)	67.0 (59.3–85.0)	-
**Age at diagnosis (years)**	1.0 (0.6–4.0)	0.9 (0.6–2.2)	1.0 (0.6–4.0)	11.9 (1.1–24.3)	-
**Height (cm)**	145.0 (132.0–154.0)	135.0 (117.5–143.0)	160.0 (152.0–162.0)	152.0 (149.8–154.0)	-
**BMI (kg/m^2^)**	25.1 (20.7–29.0)	21.5 (19.2–27.0)	29.0 (25.1–29.6)	27.7 (24.3–35.8)	Children: ≥p3- <p85; Adults: 18.5 to 24.9.
**Treatment duration (years)**	9.4 (5.8–15.4)	8.5 (6.2–10.4)	15.4 (9.5–15.5)	12.3 (2.5–20.8)	-
**UCCS (g/kg/day)**	6.3 (4.5–8.0)	8.0 (4.8–10.9)	5.3 (4.2–5.6)	5.6 (3.2–6.7)	-
**Serum:**					
**-25-OH Vitamin D (ng/mL)**	26.8 (18.1–30.5)	26.1 (18.4–31.7)	28.3 (26.8–40.0)	19.9 (11.0–27.0)	Desirable: >20
**-Vitamin B12 (pg/mL)**	379.7 (289.1–561.7)	416.1 (364.3–1074.5)	263.0 (146.8–379.3)	402.1 (160.0–561.7)	>221.4
**-Calcium (mmol/L)**	2.3 (2.3–2.4)	2.4 (2.3–2.4)	2.3 (2.2–2.4)	2.3 (2.2–2.4)	2.1–2.5
**-Phosphorus (mmol/L)**	1.4 (1.2–1.6)	1.6 (1.4–1.7)	1.3 (1.2–1.5)	1.2 (1.1–1.3)	0.8–1.5
**-Glucose (mmol/L)**	4.4 (3.9–5.0)	4.5 (3.9–5.1)	4.0 (3.9–4.3)	4.6 (3.8–5.4)	4.2–5.6
-Glucose (mmol/L) *	4.6 (4.3–4.9)	4.6 (4.4–4.9)	4.4 (4.3–4.6)	4.8 (4.1–5.2)	4.2–5.6
**-Lactate (*mmol*/L)**	1.3 (1.0–2.8)	1.3 (1.1–3.5)	1.5 (1.0–1.6)	1.2 (0.9–1.8)	0.5–2.2
-Lactate (*mmol*/L) *	1.5 (1.3–2.3)	1.5 (1.2–2.2)	1.4 (1.3–1.5)	1.9 (1.5–3.4)	0.5–2.2
**-Triglycerides (mmol/L)**	2.2 (1.2–3.2)	2.2 (1.7–3.0)	2.9 (2.9–3.4)	1.3 (0.9–3.2)	< 2.3
**-Total cholesterol (mmol/L)**	4.5 (3.6–5.2)	4.5 (4.3–5.6)	4.2 (3.6–4.6)	4.6 (3.6–5.2)	<5.2
**-HDL**	0.9 (0.7–1.0)	0.8 (0.8–0.9)	0.7 (0.6–1.1)	1.0 (0.9–1.1)	>1.0
**-LDL**	2.6 (1.8–2.9)	2.7 (1.8–3.6)	1.8 (1.7–2.7)	2.5 (2.1–3.2)	<3.3
**Bone turnover biomarkers (serum)**					
**-CTX (ng/mL)**	0.7 (0.3–1.1)	1.1 (0.9–1.5)	0.5 (0.2–1.0)	0.5 (0.2–0.7)	Table S1
**-OC (ng/mL)**	55.6 (31.9–102.2)	99.9 (79.7–114.4)	48.4 (26.6–82.6)	30.2 (23.6–36.1)	Table S1
**-P1NP (µg/L)**	304.1 (75.1–420.2)	420.2 (386.0–750.7)	254.4 (139.9–337.1)	62.4 (57.3–82.1)	Table S1
**DXA—Z-score:**					
**-Whole body (minus the head)**	−0.1 (−0.9–0.20)	−0.1 (−1.0–0.3)	−0.2 (−0.9–0.0)	N/A	>−2.0
**-Proximal femur**	−0.8 (−1.0–−0.5)	N/A	N/A	−0.8 (−1.0–−0.5)	>−2.0
**-Lumbar spine**	−0.6 (−1.3–−0.2)	−0.6 (−1.3–−0.2)	−0.6 (−1.0–−0.1)	−0.7 (−1.8–−0.4)	>−2.0

Values expressed as median and interquartile range (IQR), except sex and type of GSD. F: female; M: male. UCCS: uncooked cornstarch. P: percentile. CTX: collagen type 1 C-terminal telopeptide. OC: osteocalcin. P1NP: procollagen type 1 N-terminal propeptide. LDL: low-density lipoprotein. HDL: high-density lipoprotein. N/A: not applicable. Age range: children, 0 to 11 years; adolescents, 12 to 19 years; adults, 20 years or older. * Median of the 24 months preceding DXA.

## Supplementary Materials

The following are available online at https://www.mdpi.com/article/10.3390/nu13092987/s1. Table S1: Reference intervals for biomarkers of bone turnover, Table S2: Sample profile, Table S3: Bone mineral density and biomarkers of bone turnover in patients with hepatic glycogen storage diseases, Table S4: Summary of patients with low bone mineral density, Table S5: Spearman correlations, Table S6: Intake of nutrients essential for bone metabolism among patients with hepatic glycogen storage diseases (n = 20/23), Table S7: Hepatic glycogenosis: comparison among studies that evaluated BMD using DXA.
