# Supplementary material for: Bone Mineral Density in Patients with Hepatic Glycogen Storage Diseases
Source: Nutrients. 2021 Aug 27;13(9):2987. doi: 10.3390/nu13092987 (PMC8469033; doi:10.3390/nu13092987)
Supplement: Supplementary file 1 [file nutrients-13-02987-s001.zip › nutrients-1052531-supplementary.pdf]

Supplementary Material

Table S1: Biomarkers of bone turnover – normal range values*

Age (years)	CTX (ng/mL)				P1NP (µg/L)		Osteocalcin (ng/mL)	
	Female		Male		Female	Male	Female	Male
	P2.5	P97.5	P2.5	P97.5				
0 – 1	0.404	1.529	0.354	1.199	227.2 - 4762.8	227.2 - 4762.8	20.8 - 144.3	20.8 - 144.3
1 -2	0.396	1.556	0.435	1.433	346.6 - 1088.0	346.6 - 1088.0	28.3 - 126.1	28.3 - 126.1
2- 3	0.396	1.556	0.435	1.433	178.3 - 645.7	178.3 - 645.7	30.7 - 85.4	30.7 - 85.4
3 – 4	0.396	1.556	0.435	1.433	135.2 - 746.1	135.2 - 746.1	23.9 - 98.4	23.9 - 98.4
4 – 5	0.396	1.556	0.435	1.433	85.7 - 901.7	85.7 - 901.7	22.8 - 129.3	22.8 - 129.3
5 – 6	0.396	1.556	0.435	1.433	111.5 - 768.4	111.5 - 768.4	42.1 - 128.2	42.1 - 128.2
6 – 7	0.565	1.570	0.509	1.697	187.6 - 887.3	187.6 - 887.3	30.9 - 122.2	30.9 - 122.2
7 – 8	0.565	1.570	0.509	1.697	49.9 - 1200.0	49.9 - 1200.0	12.5 - 232.5	12.5 - 232.5
8 – 9	0.565	1.570	0.509	1.697	120.4 - 1021.0	120.4 - 1021.0	25.7 - 151.1	25.7 - 151.1
9- 10	0.565	1.570	0.509	1.697	42.7 - 952.1	45.2 - 552.5	18.4 - 251.7	12.2 - 110.6
10 – 11	0.627	1.955	0.860	2.591	62.5 - 914.7	48.3 - 769.6	18.5 - 154.2	12.6 - 145.7
11 – 12	0.627	1.955	0.860	2.591	65.3 - 855.8	142.5 - 2501.7	11.9 - 140.4	31.9 - 200.9
12 – 13	0.627	1.955	0.860	2.591	47.0 - 980.9	67.2 - 854.8	13.1 - 186.7	19.8 - 164.9
13 – 14	0.627	1.955	0.860	2.591	37.1 - 1195.6	267.9 - 1514.6	16.8 - 238.9	58.7 - 236.2
14 – 15	0.312	1.104	0.540	2.035	58.5 - 451.4	148.1 - 1200.0	15.4 - 88.8	25.7 - 241.0
15 – 16	0.312	1.104	0.540	2.035	45.6 - 600.2	81.8 - 961.4	16.9 - 96.3	30.1 - 186.9
16 – 17	0.312	1.104	0.540	2.035	14.6 - 238.3	77.7 - 430.3	5.7 - 66.7	32.0 - 124.2
17 – 18	0.312	1.104	0.540	2.035	36.3 - 143.9	38.7 - 494.5	20.7 - 45.6	13.5 - 160.1
Adults	<0.650		<0.850		13.8 - 60.9	13.9 - 85.5	11 - 48	11 – 48

P: percentile. P1NP: procollagen type 1 N-terminal propeptide. CTX: collagen type 1 C-terminal telopeptide.

Reference: Bayer et al. 2014; Wyness et al. 2013. * in the current study, we considered abnormal only values 20% above the upper or the lower limit of the reference ranges, respectively.

Table S2: Body mass index, treatment and biochemical findings of patients with hepatic glycogen storage diseases (n= 23)

Patient	Gender	Age (years)	GSD	Body mass index	Treatment duration (years)	Dietary supplementation	Glucose (mmol/L)	Lactate (mmol/L)	Triglycerides (mmol/L)	Total cholesterol (mmol/L)
1	F	6.6	Ia	Overweight	5.8	Multi	4.6	5.8	1.8	4.5
2	F	8.0	Ia	Obesity	6.6	Multi, Ca	4.6	3.3	3.0	4.5
**3**	**M**	**10.9**	**Ia**	**Obesity**	**10.6**	**Multi, Ca**	**5.1**	**1.5**	**2.1**	**4.3**
4	M	11.6	Ia	Overweight	10.9	Multi	4.2	1.5	2.7	6.6
5	M	11.8	Ia	Obesity	6.8	Multi, Ca	5.4	2.4	8.6	6.7
6	F	13.1	Ia	Obesity	6.0	Multi, Ca	4.3	1.4	2.9	5.1
7	F	13.5	Ia	Overweight	9.5	Multi, D, Ca	4.7	2.3	3.8	3.6
8	F	19.2	Ia	Eutrophic	18.6	Multi, D, Ca	4.1	1.3	3.4	4.6
**9**	**F**	**20.9**	**Ia**	**Obesity**	**20.8**	**Ca, D**	**4.8**	**1.5**	**1.7**	**5.2**
**10**	**F**	**21.2**	**Ia**	**Obesity**	**20.4**	**Ca, D**	**5.2**	**1.7**	**0.7**	**3.6**
11	F	24.5	Ia	Eutrophic	2.5	Multi, Ca	5.2	3.4	3.2	5.7
12	F	30.4	Ia	Overweight	0.2	N	3.2	4.7	4.6	4.5
13	M	10.4	Ia	Obesity	9.4	Multi, Ca	4.7	1.1	3.7	5.6
**14**	**F**	**3.5**	**Ib**	**Overweight**	**3.0**	**Multi**	**4.5**	**1.9**	**1.7**	**2.9**
**15**	**M**	**5.1**	**Ib**	**Obesity**	**2.5**	**Multi, Ca**	**4.3**	**1.9**	**2.2**	**5.6**
**16**	**M**	**10.8**	**Ib**	**Overweight**	**10.3**	**Multi**	**4.9**	**0.8**	**0.6**	**2.5**
17	M	15.9	Ib	Obesity	15.4	Multi, Ca	4.4	1.5	2.9	3.6
**18**	**F**	**33.6**	**Ib**	**Overweight**	**31.7**	**Multi**	**4.8**	**2.1**	**0.9**	**3.4**
**19**	**F**	**11.9**	**IIIa**	**Eutrophic**	**11.3**	**Ca**	**5.0**	**1.4**	**2.2**	**5.1**
**20**	**M**	**16.4**	**IIIa**	**Obesity**	**15.5**	**Multi, Ca**	**4.6**	**0.6**	**1.2**	**4.2**
21	M	11.3	IXα	Eutrophic	9.3	Multi, Ca	4.4	1.4	2.3	-
**22**	**M**	**11.7**	**IXα**	**Eutrophic**	**7.7**	**N**	**4.8**	**0.9**	**0.5**	**4.4**
**23**	**M**	**29.2**	**IXα**	**Eutrophic**	**4.2**	**N**	**4.1**	**0.6**	**0.9**	**4.7**

F: female. M: male. Supplementation: Multi: multivitamin; D: vitamin D; Ca: calcium; N: none. -: No information. In bold, patients classified as adherent to treatment.

Lactate and glucose values refer to the median of the 24 months preceding DXA. Triglycerides and total cholesterol refer to the closest measurement to DXA. Patients with normal lactate, triglyceride and glucose concentrations were considered adherent to treatment.

Table S3: Bone mineral density and biomarkers of bone turnover in patients with hepatic glycogen storage diseases (n= 23)

Patients		DXA						Biomarkers bone turnover		
n	Fractures	Lumbar		Femur		Whole body less head		CTX	P1NP	OC
		g/cm^2	Z-score	g/cm^2	Z-score	g/cm^2	Z-score	ng/mL	mcg/L	ng/mL
**1**	**No**	**0.491**	**-2.2**	**N/A**	**N/A**	**0.545**	**-2.1**	**-**	**356.7**	**-**
2	No	0.773	1.0	N/A	N/A	0.733	0.5	-	-	-
3	No	0.695	-0.6	N/A	N/A	0.330	-1.0	-	-	-
4	Yes*	0.716	-0.6	N/A	N/A	0.794	-0.1	1.070	389.8	93.5
5	No	0.764	-0.2	N/A	N/A	0.834	0.3	0.461	234.9	69.3
6	No	1.113	1.1	N/A	N/A	1.029	1.6	0.774	370.2	63.0
7	No	0.887	-1.0	N/A	N/A	0.837	-0.9	-	-	-
8	No	1.110	-0.6	N/A	N/A	0.928	-1.3	0.273	75.1	33.9
9	No	1.087	-0.8	0.896	-0.9	N/A	N/A	0.599	82.1	31.9
10	No	1.137	-0.4	0.953	-0.5	N/A	N/A	0.174	60.5	23.6
11	No	0.958	-1.8	0.879	-1.0	N/A	N/A	0.945	124.2	42.8
12	No	1.114	-0.6	0.91	-0.8	N/A	N/A	0.070	22.6	12.8
13	No	0.650	-1.0	N/A	N/A	0.763	-0.1	1.440	530.3	129.8
14	No	0.513	N/A	N/A	N/A	0.549	-	1.710	826.5	111.2
15	No	0.513	-1.3	N/A	N/A	0.608	0.1	1.070	856.9	117.7
16	No	0.788	0.2	N/A	N/A	0.918	0.9	-	-	-
17	No	1.121	-0.1	N/A	N/A	1.018	0.0	0.094	204.8	19.4
18	No	0.947	-1.9	0.83	-1.3	N/A	N/A	0.342	64.4	36.1
**19**	**No**	**0.643**	**-2.3**	**N/A**	**N/A**	**0.666**	**-2.1**	**1.070**	**386.0**	**48.2**
**20**	**No**	**0.893**	**-2.0**	**N/A**	**N/A**	**1.024**	**-0.2**	**1.220**	**304.1**	**102.2**
21	No	0.727	-0.4	N/A	N/A	0.788	-0.1	0.750	420.2	90.2
22	No	0.768	-0.2	N/A	N/A	0.818	0.1	1.670	750.7	106.3
23	No	1.187	-0.3	1.06	-0.2	N/A	N/A	0.699	57.3	28.5

N/A: Not applicable; -: Data not collected.
CTX: collagen type 1 C-terminal telopeptide. OC: osteocalcin. P1NP: procollagen type 1 N-terminal propeptide.
*Fracture after fall from height.
Bold: patients have abnormal BMD. Underlined: Bone turnover markers above the NRV.

Table S4: Summary of patients with low bone mineral density (n = 3)

**Patient**	**1**	**19**	**20**
GSD	Ia	IIIa	IIIa
Gender	F	F	M
Age (yr)	6	11	16
Height (cm, Z-score)	108 (**<-2)**	134 (**<-2)**	165 (>-2 and <-1)
BMI (kg/m^2)	**18.0**	21.6	**29.0**
Good metabolic control	No	Yes	Yes
Age at onset of treatment (mo)	8	6	12
Blood:			
- Vitamin D (ng/mL)	30.5	21.7	24.7
- Vitamin B12 (pg/mL)	**1456.0**	—	—
- Calcium (mmol/L)	2.3	2.2	2.3
- Phosphorus (mmol/L)	**1.9**	—	1.4
- Glucose (mmol/L)*	4.6	5.0	4.6
- Glucose (mmol/L)	4.4	**6.6**	5.0
- Lactate (mmol/L)*	**5.78**	1.1	0.6
- Lactate (mmol/L)	**3.8**	0.9	0.7
- Triglycerides (mmol/L)	1.8	2.2	1.2
- Total cholesterol (mmol/L)	4.5	5.1	4.2
- HDL	1.0	**0.5**	**0.6**
- LDL	2.7	**3.6**	2.9
Food record data:			
- Energy intake (kcal)	**1635.6**	—	**2308.7**
- Protein (g)	52.7	—	**73.3**
- Vitamin B6 (mg)	**1.3**	—	**2.0**
- Vitamin B12 (µg)	**2.0**	—	2.3
- Vitamin D (µg)	**2.7**	—	**30.0**
- Vitamin K (µg)	**24.0**	—	**6.0**
- Calcium (mg)	**193.7**	—	**176.7**
- Phosphorus (mg)	**608.0**	—	**649.7**
- Potassium (mg)	**1241.3**	—	**1247.0**
Supplemental intake:			
- Energy intake (kcal)	0.0	0.0	0.0
- Protein (g)	0.0	0.0	0.0
- Vitamin B6 (mg)	0.2	0.0	1.5
- Vitamin B12 (µg)	0.6	1.0	3.0
- Vitamin D (µg)	80.0	0.0	280.0
- Vitamin K (µg)	0.0	0.0	65.0
- Calcium (mg)	0.0	500.0	750.0
- Phosphorus (mg)	0.0	0.0	0.0
- Potassium (mg)	0.0	0.0	0.0

—: Data not collected. F: female. M: male. GSD: glycogen storage disease. BMI: body mass index. LDL: low-density lipoprotein. HDL: high-density lipoprotein.
*Median of the 24 months preceding DXA.
Bold face denotes above-adequate values; bold + underline denotes below-adequate values. Food record data: (dietary intake + prescribed supplementation). The daily energy requirement was calculated using the Harris Benedict formula, which takes weight, structure, age, in addition to the degree of physical activity (male: REE = 66.47 + 13.75 3 Wt + 5.0 3 Ht 2 6.76 3 age; female: REE = 655.10 + 9.56 3 Wt + 1.85 3 Ht 2 4.68 3 age), the degree of physical activity was considered mild in all patients: 1.4 - 1.59. Adequacy was considered if values were between 90 and 110% of expected.

Table S5: Spearman correlations

Correlation	N	r	p
CTX vs			
- Age	18	-0.677	0.002
- BMI	18	-0.568	0.014
- P1NP	18	0.814	<0.001
- Weight	18	-0.652	0.003
P1NP vs.			
- Age	19	-0.909	< 0.001
-Weight	19	-0.690	0.001
Osteocalcin vs.			
- Age	18	-0.766	0.001
- Weight	18	-0.668	0.002
Adequacy of Kcal intake vs.			
- CTX	15	-0.686	0.005
- Osteocalcin	15	-0.761	0.001
- P1NP	16	-0.776	0.001

Moderate and strong correlations with n ≥ 15 (65% of the sample). Significant at $p \leq 0.004$ (Bonferroni correction for multiple comparisons).

Table S6: Intake of nutrients essential for bone metabolism among patients with hepatic glycogen storage diseases (n= 20/23)

	Children (n=11)			Adolescents (n = 5)			Adults (n = 4)		
	Ia (n = 6)	Ib (n = 3)	IXα (n = 2)	Ia (n = 3)	Ib (n = 1)	IIIa (n = 1)	Ia (n = 2)	Ib (n = 1)	IXα (n = 1)
**Energy (kcal)**	2113.2 ± 160.4	2195.3 ± 279.2	1791.5	2689.7 ± 257.2	2481.7	2308.7	3219.5	2372.7	2983.0
**Cornstarch (g/kg/day)**	6.4 ± 1.1	10.9 ± 1.1	5.8	5.3 ± 1.1	5.6	4.2	6.7	4.8	1.7
**Protein (kcal)**	61.2 ± 7.5	75.7 ± 10.0	58.7	103.7 ± 13.7	48.7	73.3	69.8	74.7	133.0
**Vitamin B6 (mg)**	2.2 ± 0.3	2.0 ± 0.2	0.9	3.0 ± 0.4	2.6	3.6	2.7	3.6	1.7
**Vitamin B12 (mcg)**	5.9 ± 0.8	3.6 ± 0.8	1.6	8.4 ± 1.4	3.1	5.3	2.4	9.8	4.3
**Vitamin D (IU)**	227.3 ± 76.9	234.7 ± 7.8	70.3	699.3 ± 90.7	201.0	310.0	404.3	213.0	152.3
**Vitamin K (mcg)**	93.9 ± 18.3	20.0 ± 24.1	1.3	113.0 ± 36.5	117.0	71.0	65.0	72.0	9.0
**Calcium (mg)**	709.7 ± 118.5	427.7 ± 303.5	405.0	905.0 ± 175.1	1119.0	926.7	650.0	289.0	789.3
**Phosphorus (mg)**	649.5 ± 64.4	746.7 ± 119.6	710.7	886.7 ± 135.8	561.3	774.7	665.0	794.7	1491.0
**Potassium (mg)**	1098.0 ± 154.9	1194.0 ± 91.7	1443.5	1423.7 ± 342.7	783.3	1247.0	1537.7	1278.7	3160.0

Data refer to both food intake, obtained from the 3-day food diary, and supplementation.

Values expressed as median and standard error, except for adolescents and adults with GSD Ib, IIIa and IXα (given as mean).

N=20 (Ia:11; Ib: 5; III: 1; IXα: 3). 3 patients did not complete the food diary. Age range: children, 0 to 11 years; adolescents, 12 to 19 years; adults, 20 years or older.

Table S7 - Hepatic Glycogenosis: comparison among studies that evaluated BMD using DXA

Reference	Country	Sample (n, type GSD)	Age (y)	BMI (kg/m²)	Treatment	DXA			Biomarkers bone turnover		Biochemical/ nutritional Biomarkers	Criteria for adherence-metabolic control
						Skeletal sites/equipment	Diagnostic criteria	BMD findings	Types	Results		
Rake et al. (2003)	The Netherlands	29 GSD Ia<br>Prepubertal=8<br>Adolescents=12<br>Adults=9<br>Not controlled.	15.7 (median)	NA	Galactose and fructose intake restricted (all patients). Frequent meals during the daytime and:<br>-GDF overnight (n=4);<br>-UCCS during the daytime and continuous GDF overnight (n= 19);<br>-UCCS during the daytime and overnight (n= 6).	Lumbar spine. DXA = Hologic QDR 1000, Hologic, Inc., Waltham, MA, USA.	**Low BMD**<br>Z-score ≤2.**0**	**Low BMD**<br>Adolescents=12/12<br>Adults=9/9	NA	NA	Cholesterol negatively correlated with BMD. BMD does not depend on the metabolic control.	Plasma cholesterol and triglycerides, and urinary excretion of lactate.<br><br>**Optimal / Intermediate / Non-optimal:**<br>Prepubertal= 1 / 4 / 3<br>Adolescents= 4 / 6 / 2<br>Adults= 2 / 4 / 3
Cabrera Abreu et al. (2004)	United Kingdom	14 GSD (I=6; III=4; IX=4)**.<br>Not controlled.	I=21.5<br>III=31.1<br>IX=31.1<br>(means)	NA	Gastrostomy (I=1); Protein supplementation (III=1); Protein, calcium and vitamin supplementation (III=1); Normal diet (I=5; III=2; IX=4).	Whole body (minus the head); spine; right and left hips.<br>DXA= Lunar DPX-L pencil beam densitometer, GE Medic al Systems, Waukesha, Wisconsin, USA, Software 13Z.	**Low BMD**<br>Z-score -1 to -2.5<br>**Very low BMD**<br>Z-score <-2.5	**Very low BMD**<br>I=1/6<br>**Low BMD**<br>I=5/6<br>III=2/4<br>IX=1/4	PICP; PINP; CTX; bone-specific ALP (serum).	No consistent pattern either within or between GSD groups. No correlation between any markers of bone and whole body (minus the head) BMD Z-score. The patient with the lowest whole body (minus the head) BMD Z-score	NA	NA

										showed the highest ratios of both urinary fPYD: creatinine and fDPD: creatinine.		
Mundy et al. (2008)	United Kingdom	15 GSD III (IIIa=12; IIIb=3). 15 control subjects.	IIIa=23.6 IIIb=26.5 (means)<br><br>Mean difference controls and patients: 1.49, p=0.05.	IIIa=23.5 IIIb=22.3 (means)	Not clearly described in the manuscript.	**Adults**: Whole body (minus the head); lumbar spine; both hips. **Children:** Whole body (minus the head); lumbar spine;<br><br>DXA = GE Lunar Prodigy, GE Healthcare, Hertfordshire, UK.	**Low BMD** Z-score <-2.0	**Low BMD** 40%patients for whole body (minus the head), 64% for lumbar spine and 11% for hip. GSD IIIa patients showed lower bone mass for the whole body (minus the head) and lumbar spine than GSD IIIb patients.	NA	NA	No differences in the plasmatic calcium, 25(OH)D, ALP or PTH levels when compared with control subjects.	NA
Minarich et al. (2012)	USA	54 GSD I (Ia=42; Ib=12) Not controlled.	Ia=28.8 Ib= 29.7 (medians)	NA	Not clearly described in the manuscript.	Lumbar spine and either the left hip or whole body (minus the head) No details about bone scan method.	**Low BMD** Z-score <-2.0	**Low BMD** Ia=23/42 Ib=8/12	NA	NA	GSD Ia: mean serum 25(OH)D level was higher in patients with low BMD than in patients with normal BMD.	NA
Melis et al. (2014)*	Italy	38 GSD I (Ia=29; Ib=9). 58 control subjects.	Ia=11.4 Controls= 12.1 Ib=14.2 Controls= 17.8 (means)	Ia=22.1 Controls = 21.0 Ib=24.3 Controls=24.1 (means)	UCCS during the day, and nocturnal GDF (Ia=22; Ib=2); Only UCCS (Ia=7; Ib=7). UCCS dosage	Lumbar spine DXA = Hologic QDR 1000; Hologic Inc., Waltham, Mass., USA.	**Osteopenia** Z-score <-1.0 **Osteoporosis**: Z-score < -2.5	**DXA Z-score** Ia= -1.4 Ib= -1.7 (means)	Serum CTX; urinary NTX and hydroxyproline; OC.	Increased levels of CTX, NTX and hydroxyproline in patients.	Calcitonin was positively correlated with insulin and inversely correlated with PTH in GSD Ia . DXA	Mean values of serum glucose, lactate, uric acid, triglycerides, insulin and free IGF-I over a period of 5

					ranged between 1-1.5 g/kg/meal.						Z-score was correlated with lactic acid (r=-0.89, p=0.03) and insulin (r= 0.67, p=0.04).	consecutive years before enrollment.<br><br>**Good / Poor adherence**<br>Ia= 14 / 15<br>Ib= 7 / 2
Melis et al. (2016)*	Italy	9 GSD III (IIIa=4; IIIb=5). 18 control subjects.	IIIa=12.5<br>IIIb=7.5<br>(medians)<br><br>Controls matched to age and sex.	No significant differences in BMI were observed between patients and controls.	UCCS during day and night for all patients; Protein supplementation (4 patients). The dose of UCSS ranged between 0.50-0.80 g/kg/meal.	Lumbar spine. DXA = Hologic QDR 1000; Hologic Inc., Waltham, Mass., USA.	**DXA:**<br>Z-scores reference values according to the manufacturer's internal reference database.	The patients presented a **lower** DXA Z-score (−1.21 vs 0.74, p=<0.05) and QUS (−2.10 vs 0.13, p=<0.05) when compared with controls.	ALP; CTX; OC (serum).	Patients had lower levels of OC and increased levels of CTX than controls.	GSD III patients showed decreased levels of calcitonin. DXA Z-score is inversely correlated with total cholesterol levels and triglycerides.	NA
Kaiser et al. (2019)	Switzerland	25 GSD I (Ia=22; Ib=3) No control subjects.	Ia=19<br>Ib=22<br>(medians)	Ia=21.7<br>Ib=25.2<br>(medians)	Regular carbohydrate intake.<br>-UCCS during daytime (17 patients).<br>**Type of night-time nutrition for adults:**<br>UCCS=12<br>GDF=4<br>**Type of night-time nutrition for children:**<br>UCCS=2<br>GDF=7	Lumbar spine; Hip.<br>No details about DXA machine in the manuscript.	**Low BMD**<br>Z-score <-2.0	**Low BMD**<br>Ia=6/22<br>Ib=2/3<br><br>Mean Z-scores were lower at the lumbar spine compared to the hip.	NA	NA	Z-score correlated negatively with serum lactate in GSD Ia patients presenting normal bone age.	NA
This study	Brazil	23 GSD I (Ia=13; Ib=5; IIIa=2; IXα= 3).	Ia=13.1<br>Ib=10.8<br>IIIa=14.2<br>IXα=11.7	Ia=27.6<br>Ib=21.9<br>IIIa=25.3<br>IXα=19.1	UCCS 4 to 8 times a day for all patients; median dose 6.3	**Adults:**<br>Lumbar spine; proximal femur.	**Low BMD**<br>Z-score ≤-2.0	**Low BMD**<br>Ia=1/13<br>IIIa=2/2<br>**Normal BMD**	CTX; P1NP; OC (serum).	CTX, OC and P1NP correlated negatively	No correlations between biochemical	Serum triglyceride (the measurement closest to the

	No control subjects.	(medians)	(medians)	g/kg/day. The diet for GSD I patients includes restriction of sucrose, fructose, galactose and lactose. For types III and IX, the restriction of sucrose and high-protein diet is recommended	**Children:**<br>Lumbar spine; whole body (minus the head) .<br>DXA = Lunar Prodigy Primo device; Encore version 14.1, Radiation Corporation, Madison, WI.	Ia=12/13<br>Ib=5/5<br>IIIa=0/2<br>IXα=3/3	both with age and body weight.	biomarkers and BMD. Phosphorus, calcium, and potassium intakes were below the dietary requirements, even after nutritional supplementati on intake	date of DXA), glucose and lactate concentrations (median value of the previous 24 months-measurements).<br>**Good / Poor adherence**<br>Ia=3 / 10<br>Ib=4 / 1<br>IIIa=2 / 0<br>IXα=2 / 1

GSD: Hepatic glycogen storage diseases. Y: years. BMI: Body mass index. DXA: Dual-energy X ray absorptiometry. BMD: Bone mineral density. GDF: *gastric drip feeding. UCCS:* uncooked cornstarch. PICP: Procollagen Type I C-terminal Propeptide. *P1NP:* procollagen type 1 N-terminal propeptide. CTX: collagen type 1 C-terminal telopeptide. fPYD: Free pyridinoline. Fdpd: Free deoxypyridinoline. ALP: alkaline phosphatase. PTH: parathyroid hormone. USG: Ultrasonography. NTX: N-terminal telopeptides. IGF: insulin-like growth factor. OC: Osteocalcin. QUS: quantitative ultrasound. 25(OH)D: 25-OH vitamin D.

* = BMD was also evaluated using quantitative ultrasonography. Lateral surfaces of the fingers (II–V; nondominant hand) in the proximity of the condyles QUS = DBM Sonic 1,200 apparatus; IGEA, Carpi, Italy. Z score, on the basis of the standards provided by the manufacturer. ** = subtypes were not identified by the authors. NA = not available.
